# Dioscin Inhibits the Invasion and Migration of Hepatocellular Carcinoma HepG2 Cells by Reversing TGF-β1-Induced Epithelial-Mesenchymal Transition

**DOI:** 10.3390/molecules24122222

**Published:** 2019-06-14

**Authors:** Bonan Chen, Shikun Zhou, Yujuan Zhan, Junzi Ke, Kun Wang, Qiqi Liang, Yu Hou, Pingping Zhu, Weizhen Ao, Xianli Wei, Jianyong Xiao

**Affiliations:** 1 Department of Biochemistry, Guangzhou University of Chinese Medicine, Guangzhou 510006, China; 20171104455@stu.gzucm.edu.cn (B.C.); 20161104381@stu.gzucm.edu.cn (Y.Z.); 2 Research Center of Integrative Medicine, School of Basic medicine, Guangzhou University of Chinese Medicine, Guangzhou 510006, China; 2016101032@stu.gzucm.edu.cn (S.Z.); 2016101055@stu.gzucm.edu.cn (J.K.); liangqiqi13@163.com (Q.L.); 2015041017@stu.gzucm.edu.cn (Y.H.); 2015041037@stu.gzucm.edu.cn (P.Z.); a1758422883@163.com (W.A.); 3 Department of Pathology, Guangzhou University of Chinese Medicine, Guangzhou 510006, China; 20172104085@stu.gzucm.edu.cn; 4 Department of Medical Instruments, Guangdong Food and Drug Vocational College, Guangzhou 510006, China

**Keywords:** dioscin, epithelial-mesenchymal transition, hepatocellular carcinoma cells, TGF-β1, MAPK

## Abstract

Dioscin is a natural steroidal saponin that can be isolated from Chinese medicine, such as Dioscoreae rhizoma. It has wild range of pharmacological activities such as hepatoprotection, a lipid-lowering effect, and anti-inflammation. Recently, mounting studies reported the anticancer effect of dioscin on a variety of tumor cells. However, the potential effect of dioscin on the epithelial-mesenchymal transition (EMT) of HepG2 cells is unclear. In the present study, dioscin was identified to inhibit transforming growth factor-β1 (TGF-β1) and induced invasive and migratory behavior of HepG2 cells. Consistently, the expression of the epithelial marker E-cadherin and gap junction proteins increased following dioscin treatment, while mesenchymal markers decreased, including N-cadherin, Vimentin, Snail, and Slug. Furthermore, we discovered that TGF-β1 induces phosphorylation of JNK, p38, and Erk, whereas the activation of these kinases was reversed by dioscin treatment in a dose-dependent manner. With the addition of Asiatic acid, a p38 activator, the inhibitory effect of dioscin on EMT was reversed. Taken together, these data indicated that dioscin inhibits EMT in HepG2 cells, which is mediated in large part by inhibition of the p38-MAPK signaling.

## 1. Introduction

Dioscin is a natural steroidal saponin, one class of saponins in which the aglycone moiety is a steroid [1]. It is a derivative of diosgenin, whose position 3 was attached by a spirostanyl glycoside that consists of the trisaccharide alpha-L-Rha-(1->4)-[alpha-L-Rha-(1->2)]-beta-D-Glc via a glycosidic linkage [2]. Dioscin can be isolated from various Chinese medicines, such as Dioscoreae rhizoma, and possesses a wide range of biological activities, such as hepatoprotection, a lipid-lowering effect, and anti-inflammation [3]. Interestingly, an increasing number of studies recently reported that dioscin had an anticancer effect on a variety of cancer cells such as human leukemia K562, human lung cancer A549, and hepatocellular carcinoma Huh7 [1]. However, the effect of dioscin on the epithelial-mesenchymal transition (EMT) of HepG2 cells is still lacking.

Dioscin plays a tumor suppressive role through multiple mechanisms. For instance, dioscin induces the apoptosis of prostate cancer cells by activating estrogen receptor-β [4], dioscin induces apoptosis of gallbladder cancer by inhibiting PI3K/AKT pathway [5], and dioscin inhibits melanoma progression by upregulating connexin 43 [6]. In the present study, we discovered that dioscin can inhibit the TGF-β1-induced EMT in HepG2 cells, and the mechanisms of actions remain to be elucidated.

Hepatocellular carcinoma (HCC) often culminates in extensive metastasis and has a high recurrence rate after resection or ablation [7]. EMT occurs when epithelial cells lose cell polarity and the expression of epithelial markers, such as E-cadherin, and acquire a mesenchymal phenotype, characterized by increased expression of mesenchymal markers such as *N*-cadherin, Vimentin, and Fibronectin. Cells that have undergone EMT exhibit fibroblast-like properties and enhanced motility, and EMT is an initiating step in the metastasis of epithelial cancers [8,9]. 

Transforming growth factor-β1 (TGF-β1) can induce EMT and promote the development of a mesenchymal phenotype by inducing expression of EMT markers. TGF-β1-stimulated cells become spindle-shaped and undergo morphological changes, including a decrease in cell-cell adhesion and loss of polarity [10]. TGF-β1 activates many signaling pathways, including the mitogen activated protein kinase (MAPK) pathways, which play key roles in promoting EMT and metastasis [11].

Here, we demonstrate that dioscin is a potent inhibitor of the TGF-β1-induced EMT in HepG2 cells, and we evaluate potential mechanisms by which dioscin inhibits TGF-β1-induced EMT.

## 2. Results 

### 2.1. Dioscin Inhibits Proliferation of HepG2 Cells

**Figure 1:**
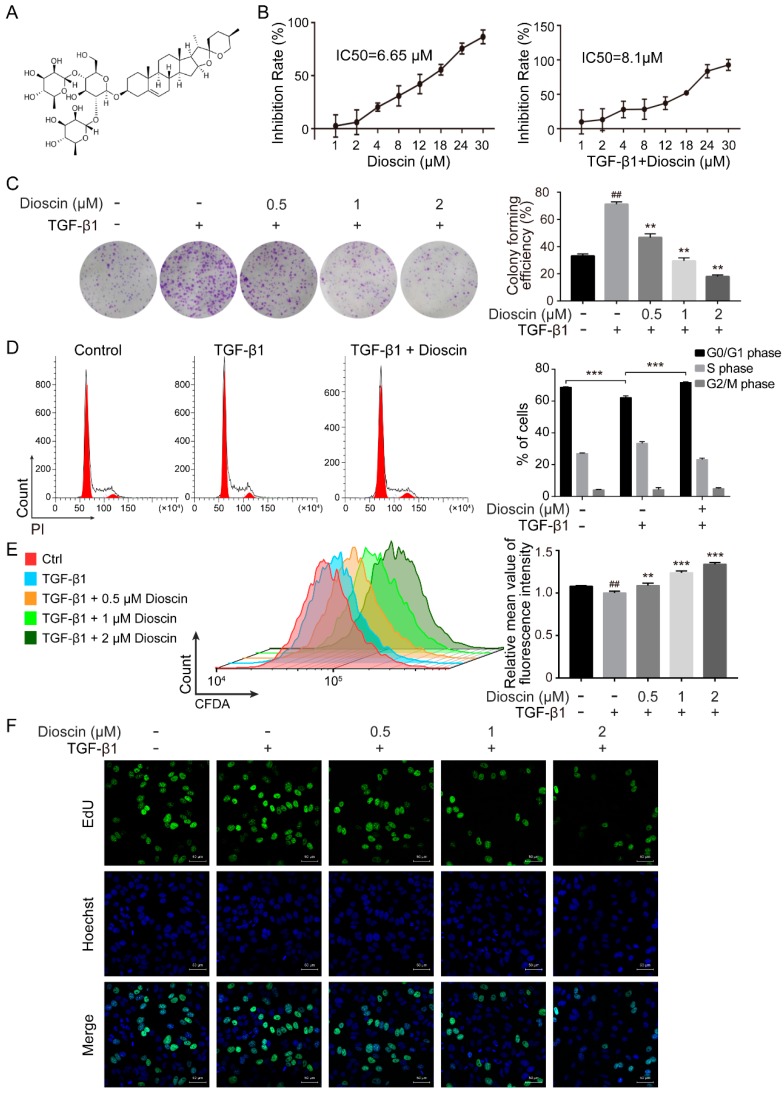
Dioscin inhibits the proliferation of HepG2 cells. (**A**) The molecular structure of dioscin. (**B**) Dioscin inhibits the viability of HepG2 cells, as determined by the CCK8 assay. The inhibitory concentration (IC50) was calculated using GraphPad Prism 6.0 software. (**C**) Dioscin inhibits the colony formation of HepG2 cells. (**D**) Cell cycle distribution of HepG2 cells treated with dioscin was analyzed by flow cytometry. (**E**) Dioscin inhibits the division of HepG2 cells. Drug-treated cells were stained by CFDA-SE and analyzed by flow cytometry. (**F**) Dioscin inhibits the DNA synthesis of HepG2 cells. Drug-treated cells were labeled with EdU. The EdU-positive cells are marked in green; Hoechst 33,342 (blue) was used for nuclear staining. Images were acquired using a confocal laser scanning microscope. The scale bar represents 50 μm. Cells were treated with 5 ng/mL TGF-β1 for 24 h. ## *p* < 0.01 vs. control group; ** *p* < 0.01 and *** *p* < 0.001 vs. TGF-β1-induced group.

We assessed the cytotoxicity of dioscin to HepG2 cells using a CCK-8 assay. We found that dioscin decreased the viability of HepG2 cells in a dose-dependent manner with a half maximal inhibitory concentration (IC50) of 6.65 µM dioscin (Figure 1B). Following induction with TGF-β1, the IC50 of dioscin for HepG2 cells increased to 8.1 µM (Figure 1B). Because we aimed to examine the effects of dioscin on metastasis of HepG2 cells, we selected the less cytotoxic concentrations of dioscin (0.5, 1, 2 µM) to treat HepG2 cells for subsequent experiments. Low concentration of dioscin reversed the growth-promoting effect of TGF-β1 on HepG2 cells in a colony formation assay (Figure 1C). Cell cycle analysis demonstrated that TGF-β1 induced an increase in *S*-phase HepG2 cells concomitant with a decrease in G0/G1 phase cells, and this effect was rescued by treatment with dioscin (Figure 1D).

To further determine the effect of dioscin on cell division, HepG2 cells were stained with CFDA-SE. When cells divide, the fluorescence intensity of CFDA-SE decreases because the labeling dye is distributed equally to daughter cells. Dioscin treatment increased the CFDA-SE fluorescence intensity, suggesting that dioscin inhibited HepG2 cell division (Figure 1E). The inhibitory effects of dioscin on DNA synthesis were also confirmed by a 5-ethynyl-2′-deoxyuridine (EdU) incorporation assay. The number of EdU-positive HepG2 cells after dioscin treatment was significantly decreased compared to the control group (Figure 1F). These results indicate that low concentrations of dioscin antagonize the TGF-β1-induced proliferation of HepG2 cells.

### 2.2. Dioscin Inhibits the Migration and Invasion of HepG2 Cells

**Figure 2:**
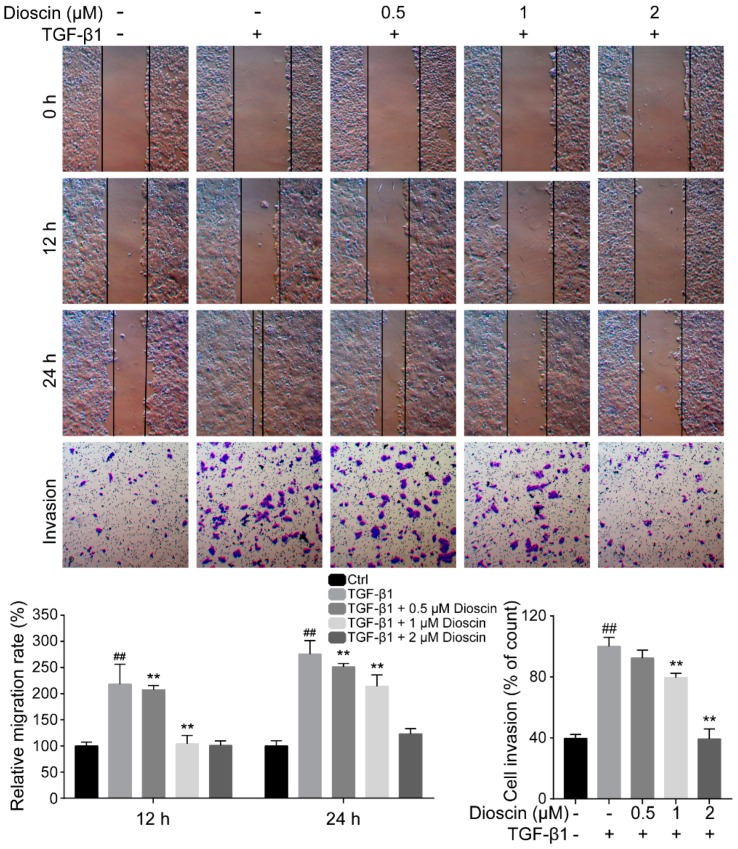
Dioscin inhibits the migration and invasion of HepG2 cells. The migration of HepG2 cells was examined using wound healing assays; the invasion of HepG2 cells was determined by transwell assays. Cells were treated with 5 ng/mL TGF-β1 for 24 h. ## *p* < 0.01 vs. control group; ** *p* < 0.01 vs. TGF-β1-induced group.

Due to the low metastatic propensity of HepG2 cells, we treated HepG2 cells with TGF-β1, a well-established inducer of metastatic behavior. HepG2 cells treated with 5 ng/mL TGF-β1 showed elevated migration and invasion (Figure 2). Dioscin treatment significantly inhibited TGF-β1-induced migration of HepG2 cells (Figure 2). Consistently, the transwell invasion assay demonstrated that dioscin treatment also inhibited the invasion of HepG2 cell.

### 2.3. Effects of Dioscin on Expression of EMT Markers

**Figure 3:**
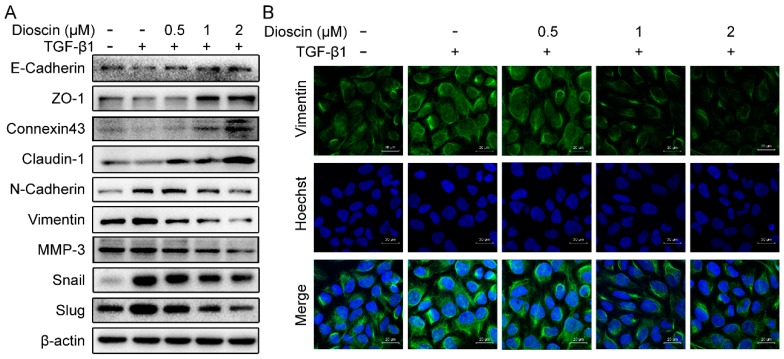
Effects of dioscin on expression of EMT markers. (**A**) Immunoblotting results. (**B**) Immunofluorescence of HepG2 cells; Vimentin is shown in green and nuclei are marked by DAPI stain. Cells were treated with 5 ng/mL TGF-β1 for 24 h.

Treatment of HepG2 cells with dioscin resulted in a significant increase in protein expression of connexin 43, ZO-1, claudin-1, and E-cadherin, compared to TGF-β1 treatment alone (Figure 3A). Furthermore, dioscin treatment reduced expression of *N*-cadherin, Vimentin, Slug, and Snail in HepG2 cells in a dose-dependent manner (Figure 3A). The decreased protein expression of Vimentin in dioscin-treated HepG2 cells was confirmed by immunofluorescence (Figure 3B).

### 2.4. Dioscin Inhibits the MAPK Pathway.

**Figure 4:**
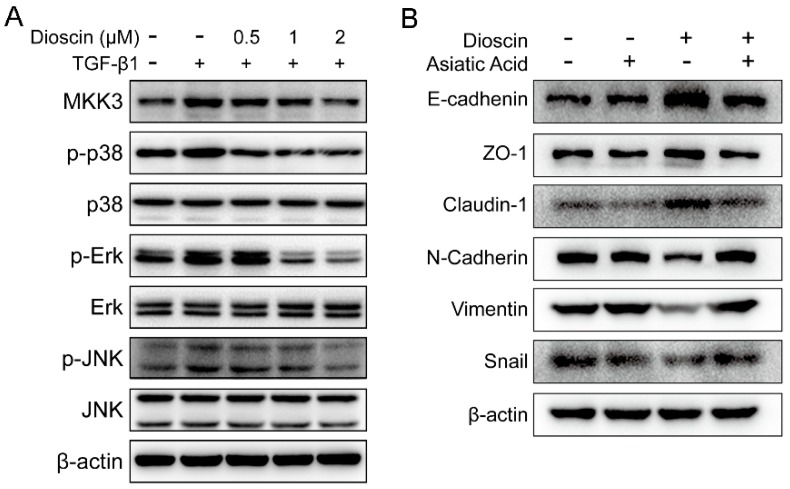
Dioscin treatment down-regulates activation of key kinases in the MAPK pathway. (**A**) The expression of the key kinases of MAPK pathway in HepG2 cells. (**B**) The expression of EMT markers in the TGF-β1-induced HepG2 cells. Cells were treated with 5 ng/mL TGF-β1 for 24 h and then with the addition of dioscin and/or 5 μM of Asiatic acid continuously cultured for additional 24 h.

The MAPK pathway plays a critical role in cancer cell growth and metastasis. To reveal the mechanisms by which dioscin inhibits the migration and invasion of HepG2 cells, we examined key kinases in the MAPK pathway. TGF-β1 induced the up-regulation of MKK3, p-Erk, p-p38, and p-JNK in HepG2 cells (Figure 4). In contrast, dioscin treatment decreased TGF-β1-induced expression of MKK3, p-Erk, p-p38, and p-JNK in a dose-dependent manner (Figure 4). The down-regulation of the phosphorylated forms of these kinases suggested that dioscin inhibits the activation of MAPK pathway in HepG2 cells. Furthermore, although dioscin could up-regulate *E*-cadhenin, ZO-1, and Claudin-1, and down-regulate N-cadherin, Vimentin, and Snail in TGF-β1-induced HepG2 cells, the actions of dioscin were reversed by Asiatic acid treatment, a p38 activator. These data suggested that the essential role of p38 and dioscin was to inhibit the EMT of HepG2 cells in large part by inhibiting p38-MAPK signaling.

## 3. Discussion

Interest has been rising in dioscin, due to its anti-cancer activities, and it has been suggested that dioscin may be a potential candidate for cancer chemotherapy [3,12]. In the present study, we report that dioscin inhibits the EMT of HepG2 cells. Furthermore, we discovered that the MAPK pathway was repressed in HepG2 cells following dioscin treatment. This finding supports the future development of dioscin as a clinical agent in cancer therapy. 

HepG2 cells are an HCC cell line commonly used to study the biology of hepatocellular carcinoma. Due to its low metastatic potential, TGF-β1 is commonly applied to induce EMT in HepG2 cells. TGF-β1 expression is a risk factor for HCC, and its abnormal expression is closely correlated with HCC tumor incidence and poor outcome [13,14]. By mimicking the paracrine inflammatory microenvironment of HCC in HepG2 cells with TGF-β1, this model can be used to study HCC EMT in vitro. TGF-β1 not only acts an inducer of cancer cell EMT, but it is also an important growth-promoting factor. HepG2 cells treated with TGF-β1 show increased growth and proliferation (Figure 1C–F). In contrast, treatment with low doses of dioscin inhibited the growth-promoting effect of TGF-β1 on HepG2 cells in a dose-dependent manner. 

EMT is an indicator of malignant transformation and is characterized by the loss of epithelial cell polarity and the acquisition of an elongated mesenchymal morphology, concomitant with the disruption of cell adhesion, increased cell migration, invasion and metastasis, and chemotherapeutic resistance [15,16,17]. Wound healing and transwell assays are commonly used to evaluate EMT, and dioscin suppressed the TGF-β1-induced migration and invasion of HepG2 cells. During EMT, epithelial cells lose their intracellular junctions, such as connexin 43, ZO-1, and claudin-1 and cohesions, and they acquire more mesenchymal properties, including increased expression of the mesenchymal markers *N*-cadherin and Vimentin, and EMT transcription factors Snail and Slug, that inhibit *E*-cadherin. Consistent with the cellular mobility analysis, our primary data showed that dioscin up-regulated the expression of *E*-cadherin and down-regulated the expression of Vimentin in HepG2 cells. Furthermore, the *E*-cadherin inhibiting transcription factors Slug and Snail were down-regulated in dioscin-treated HepG2 cells. Clinically, the loss of *E*-cadherin and increased expression of Vimentin are significantly associated with poor prognosis in a variety of cancers [18,19]. Therefore, inhibition of EMT by dioscin is a promising strategy that may prevent HCC tumor cells from additional malignant transformation.

TGF-β1-induced EMT involves signaling through the MAPK pathway [20]. The MAPK family is divided into three major subfamilies: extracellular signal-regulated kinase (Erk), c-Jun N-terminal kinase (JNK), and p38 MAPK. Inhibition of these three MAPK subfamily pathways can inhibit cancer cell metastasis [21,22]. We evaluated the effect of dioscin on the MAPK pathway and found that dioscin treatment down-regulated the expression of p-Erk, MKK-3, p-p38, and p-JNK. These data suggest that dioscin inhibits the activation of MAPK pathway downstream of TGF-β1 stimulation (Figure 4). The specific MAPK kinase that mediates the inhibitory effect of dioscin on EMT and the proliferation of HepG2 cells warrants additional study. Of note, JNK and p38 are key kinases that mediated apoptosis. Dioscin has been reported to induce apoptosis in colon cancer cells by activating JNK and p38. However, the low concentration of dioscin used in our experiments did not induce apoptosis of HepG2, but played rather inhibited the TGF-β1-triggered EMT malignant transformation, as is explained by the down-regulation of phosphorylated kinases in the MAPK pathway.

High doses of dioscin can be a double-edged sword, regardless of its ability to induce apoptosis in tumor cells, and the side effects caused by dioscin’s high cytotoxicity cannot be ignored. In addition, Smad is a well-known downstream transcription factor of TGF-β1 and involved in regulation of EMT. However, western blot analysis showed no difference in phosphoryl Smad between dioscin-treated and -untreated cells (data not shown), suggesting the inhibition of EMT by dioscin did not involve Smad. 

## 4. Materials and Methods

### 4.1. Drugs and Reagents

Dioscin (4123, 98.0% pure, MedChem Express, Princeton, NJ, USA); TGF-β1 (AF-100-21C, PeproTech, Rocky Hill, NJ, USA); and Asiatic acid (464-92-6, Selleck Chemicals, Houston, TX, USA). Primary antibodies used in this study were purchased from Cell Signaling Technology (Beverly, MA, USA): including Vimentin (5741), *N*-Cadherin (13116), Claudin-1 (13255), ZO-1 (13663), Snail (3879), Slug (9585), *E*-Cadherin (3195), Connexin-43 (3512), MMP-3 (14351), MKK3 (8535), p38 (8690), p-p38 (4511), Erk (4695), p-Erk (4370), JNK (9252), p-JNK (4668), and β-actin (3700), as well as an anti-rabbit IgG Alexa Fluor 488-conjugated secondary antibody (4412). Peroxidase-labeled antibody against mouse IgG (AS003) and peroxidase-labeled antibody against rabbit IgG (AS014) were purchased from ABclonal (Wuhan, China).

### 4.2. Cell Culture and Drug Treatments

The HepG2 cell line was obtained from the Shanghai Cell Bank (Shanghai, China). HepG2 cells were cultured in DMEM containing 10% FBS and penicillin (100 U/mL)/streptomycin (100 U/mL) at 37 °C in a humidified atmosphere with 5% CO_2_. Cells were treated with 5 ng/mL TGF-β1 for 24 h. Afterwards, the dioscin (0.5 µM, 1 µM, and 2 µM) and/or 5 μM Asiatic acid was added to the culture medium. Cells were cultured for an additional 24 h. Dioscin was dissolved in DMSO. The vehicle (DMSO solvent) was used as a control. 

### 4.3. Cell Viability Assay

Five thousand cells were plated into each well in 96-well plates and were cultured for 24 h. The cells were treated with drugs as indicated. After treatment, 100 µL CCK-8 reagent (diluted 10-fold with DMEM) was added to the culture medium of each well. Plates were incubated for 2 h in a cell culture incubator. After 2 h, the absorbance at 450 nm was measured for each well. 

### 4.4. Colony Formation Assay

HepG2 cells were plated in 6-well plates (1000 cells per well) and incubated overnight. Following drug treatment, the culture medium was replaced by fresh medium and cells were cultured for an additional 14 days. Afterwards, the cells were fixed with 4% paraformaldehyde for 20 min, washed with PBS, stained with crystal violet solution (Beyotime, Beijing, China), and photographed.

### 4.5. Cell Cycle Analysis

Cells (2 × 10^5^ cells/well) were cultured in 6-well plates. Following drug treatment, cells were collected and fixed with 70% ethanol at 4 °C overnight, then stained with a mixture containing 50 mg/mL propidium iodide and 10 mg/mL RNase A for 30 min at 37 °C. Afterwards, the cell cycle distribution was analyzed using a BD AccuriTM C6 flow cytometer (Becton–Dickinson, San Josè, CA, USA).

### 4.6. CFDA SE Cell Proliferation Assay

HepG2 cells labeled with CFDA-SE were inoculated in 6-well plates and cultured overnight. After drug treatment, cells were lifted with 0.25% (*w*/*v*) trypsin solution and collected by centrifugation (1000 rpm for 5 min). The cells were resuspended in PBS and subjected to flow cytometric analysis; fluorescence intensity was measured on a BD AccuriTM C6 flow cytometer (Becton–Dickinson).

### 4.7. 5-Ethynyl-20-deoxyuridine (EdU) Incorporation Assay

HepG2 cells were plated on a confocal dish and incubated overnight. After drug treatment, 50 μM EdU labeling agent was added to the culture medium, the cells were incubated for an additional 2 h, and then fixed with 4% paraformaldehyde for 15 min. The cells were washed with PBS, blocked with 0.5% Triton X-100 in PBS for 15 min, and then stained with anti-EdU working solution at room temperature for 30 min. Afterwards, the cells were incubated in PBS containing 5 μg/mL Hoechst 33342 at room temperature for 10 min. The cells were washed with PBS twice and then imaged under a confocal microscope LSM800 (Cal Zeiss, Göettingen, Germany).

### 4.8. Western Blot Analysis

Cells were lysed in 1× loading buffer. The samples were separated by 10% or 15% SDS-PAGE and transferred to PVDF membranes. Membranes were blocked in in a TBST buffer containing 5% skim milk for 2 h. Membranes were probed with primary antibodies and incubated overnight at 4 °C. Membranes were then washed twice with TBST and incubated in the appropriate secondary antibodies for 1 h. The resulting protein bands were visualized with enhanced chemiluminescence.

### 4.9. Wound Healing and Transwell Assays

For the wound healing assay, cells (5 × 10^5^ cells/well) were cultured in 6-well plates and pretreated with 5 ng/mL TGF-β1 for 24 h. A wound was scratched in the cell monolayer by scraping a straight line along the cell monolayer with a 200 µL pipette tip. Afterwards, culture medium was replaced with fresh medium containing dioscin (0.5 µM, 1 µM, and 2 µM) and TGF-β1 (5 ng/mL). Cells were cultured for 24 h. Cell migration was imaged at 0 h and 24 h using an inverted microscope (Olympus, Hamburg, Germany). For transwell assays, cells were treated with 5 ng/mL TGF-β1 for 24 h, transferred into the upper chamber of a matrigel-coated transwell insert (2 × 10^4^ cells/well), and supplied with 200 µL serum-free DMEM containing TGF-β1 (5 ng/mL) and dioscin (0.5 µM, 1 µM, and 2 µM), while 800 µL DMEM (15% FBS) was added to the lower chambers. After 24 h incubation, the cells that had invaded the matrigel-coated transwell insert were fixed with 4% paraformaldehyde for 15 min, stained with 0.1% crystal violet for 20 min, and imaged. The number of invaded cells was counted by Image-Pro Plus 6.0 software (Media Cybernetics, Silver Spring, MD, USA).

### 4.10. Immunofluorescence Assay

Cells (1 × 10^5^ cells/well) were seeded on glass slides in 12-well plates. After drug treatment, the cells were fixed with 4% paraformaldehyde for 20 min at room temperature and permeabilized with blocking solution (9 mL PBS, 1 mL FBS, and 30 µL Triton X-100) in a humidified and dark atmosphere for 3 h. Afterwards, cells were incubated with primary antibodies in blocking solution overnight at 4 °C, and then incubated at room temperature for 2 h with an anti-rabbit IgG Alexa Fluor 488-conjugated secondary antibody and stained with 1 µg/mL Hoechst 33342 for 10 min at room temperature. Between each step, cells were washed with PBS. The slides were sealed and immediately observed using a confocal microscope LSM800 (Carl Zeiss, Göettingen, Germany).

### 4.11. Statistical Analysis

All experiments were repeated at least three times. Data are expressed as mean  ±  S.D. One-way analysis of variance (ANOVA) and multiple comparisons were used to evaluate the statistical significance of the differences between groups. Differences were considered statistically significant at *p* < 0.05.
